# Bioimpedance spectroscopy: Is a picture worth a thousand words?

**DOI:** 10.1111/sdi.13084

**Published:** 2022-04-25

**Authors:** Maha Mohamed, Jim Matthie, Stanley L. Fan

**Affiliations:** ^1^ Department of Renal Medicine and Transplantation Barts Health NHS Trust London UK; ^2^ Matthie Consulting La Jolla CA USA

## Abstract

Volume status can be difficult to assess in dialysis patients. Peripheral edema, elevated venous pressure, lung crackles, and hypertension are taught as signs of fluid overload, but sensitivity and specificity are poor. Bioimpedance technology has evolved from early single frequency to multifrequency machines which apply spectroscopic analysis (BIS), modeling data to physics‐based mixture theory. Bioimpedance plots can aid the evaluation of hydration status and body composition. The challenge remains how to use this information to manage dialysis populations, particularly as interventions to improve over hydration, sarcopenia, and adiposity are not without side effects. It is therefore of no surprise that validation studies for BIS use in peritoneal dialysis patients are limited, and results from clinical trials are inconsistent and conflicting. Despite these limitations, BIS has clinical utility with potential to accurately evaluate small changes in body tissue components. This article explains the information a BIS plot (“picture”) can provide and how it can contribute to the overall clinical assessment of a patient. However, it remains the role of the clinician to integrate information and devise treatment strategies to optimize competing patient risks, fluid and nutrition status, effects of high glucose PD fluids on membrane function, and quality of life issues.

## 1 INTRODUCTION

Chronic volume overload measured by bioimpedance (BIA) technology is associated with hypertension, left ventricular hypertrophy (LVH), congestive heart failure and increased cardiovascular mortality in hemodialysis (HD),^1^, ^2^ and peritoneal dialysis (PD) populations.^3^, ^4^, ^5^ Volume overload occurs more frequently in PD than those receiving HD,^6^, ^7^ with a high prevalence of volume overload confirmed in studies using BIA measurements in PD patients.^8^, ^9^, ^10^ Clinical assessment of volume status can be imprecise and difficult in PD patients as they are seen less frequently and peripheral edema that is often taught as a sign of fluid overload correlates poorly with volume status.^11^ PD clinicians also avoid inducing volume depletion often used in HD to set “dry weight” in order to preserve residual renal function (RRF).

The role of BIA has been studied as a clinical tool to improve fluid management in PD.^12^ Meta‐analyses confirm that fluid overload (FO) assessed through BIA predicts mortality in PD and HD.^13^, ^14^ Will this information allow us to accurately identify overloaded patients, “dry them out,” and prevent mortality? Unfortunately, this oversimplifies the hurdles required to improve patient care.^12^ There are, in fact, limited validation studies for BIA use in PD patients, and not all BIA technologies are equivalent. It is therefore of no surprise that results from randomized controlled trials (RCTs) are inconsistent and conflicting, raising the question of whether there is any real clinical utility to the use of BIA in PD patients.^15^

While BIA may not be the complete panacea for solving issues of fluid overload in PD, this article explains how this technology has evolved and how the latest reiteration (bioimpedance spectroscopy, BIS) may make an important contribution to the overall clinical assessment of a patient. It is the role of the clinician to integrate the information and to devise treatment strategies to optimize the competing risks that a patient faces, fluid and nutrition status, the effects of using high glucose PD fluids on peritoneal membrane function, and not least, quality of life issues.

## 2 SINGLE FREQUENCY (SF) VERSUS BIS

BIA gives two main pieces of information, total tissue fluid content, equivalent to total body water (TBW), and cell mass including fat and lean tissue composition.^16^

Single frequency bioimpedance analysis (SFBIA) uses a fixed SF of 50 kHz, which passes through extracellular fluid (ECF) and some intracellular fluid (ICF).

BIS, which applies alternating current over a range of frequencies, is a well‐known powerful analytical technical in many fields of science. Much of our understanding of biological cells and tissue comes from its use in biophysics. It is used to construct physical models, so the components of a material can be analyzed separately; the response to an applied signal over a wide frequency (*f*) range provides information about the electronic circuit causing the response.

**FIGURE 1:**
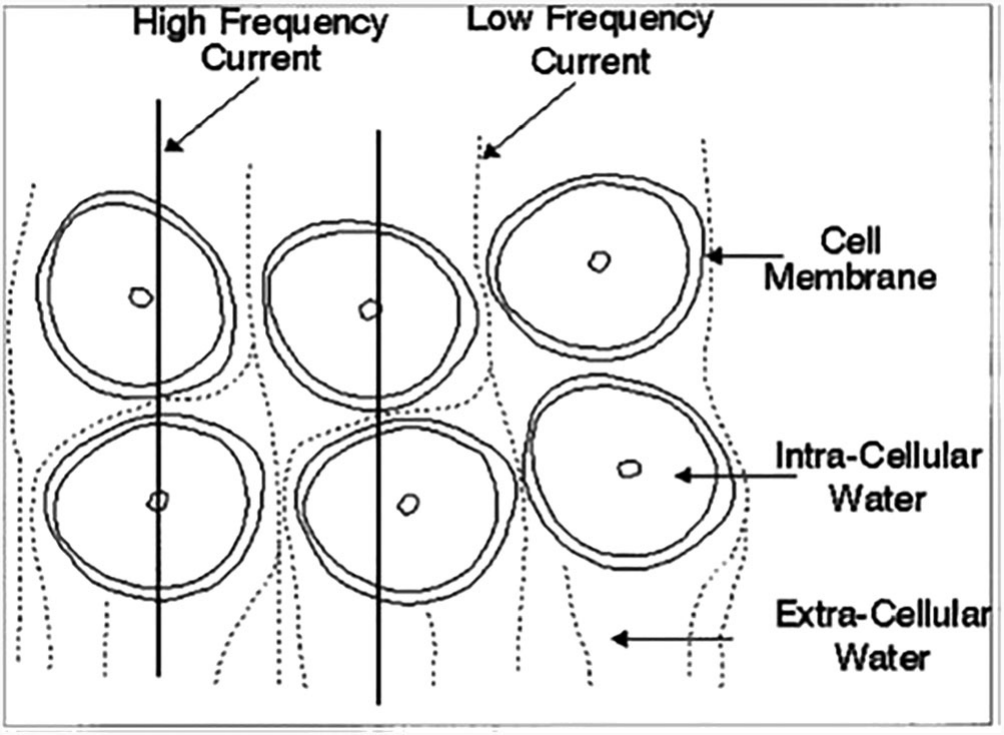
Diagram representing high‐frequency and low‐frequency current distribution in cell suspensions. With permission from the *Journal of Applied Physiology* ^17^

**FIGURE 2:**
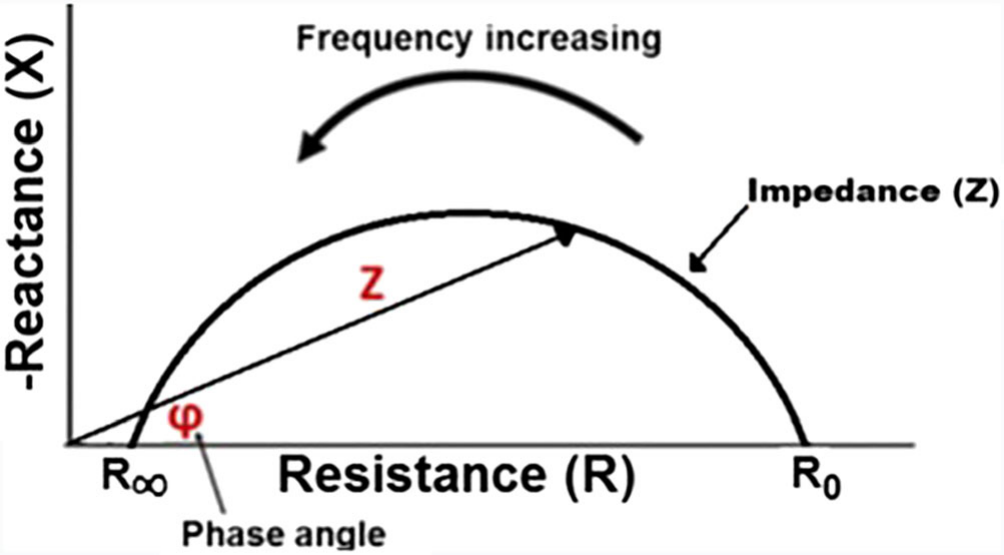
Cole–Cole impedance plot (with permission from Bojan Gavrilovic Master's thesis). This is a depiction of how reactance (*X*) and resistance (*R*) changes when the frequency of an alternating current is applied to a body [Color figure can be viewed at wileyonlinelibrary.com]

Impedance (*Z*) is the opposition to the flow of current and consists of reactance (*X*) and resistance (*R*). Resistance is a function of ionic volume (fluid), whereas *X* reflects the delay in current flow caused by cell membrane capacitance (Cm). At zero frequency (*R*_0_), there is no *X*, and the current conducts solely in the ECF. As *f* increases, more ICF is measured until the effects of Cm are insignicant. At infinite frequency (*R*_∞_ or *R*_INF_), both ECF and ICF resistances are more accurately measured.^17^ This is simplistically depicted in Figures 1 and 2.

**FIGURE 3:**
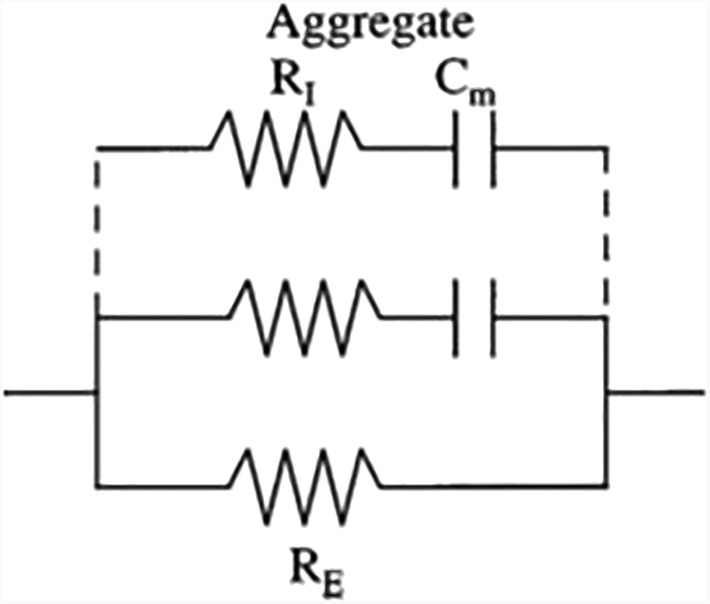
Equivalent electrical circuit analogous to Cole model. *R*_E_ is resistance ECF in ohms; *R*_I_ is aggregate resistance ICF in ohms; Cm is aggregate membrane capacitance in farads. With permission from *Journal of Applied Physiology* ^17^

In 1940, Cole developed the most widely used scientific model for studying biological cells and tissue.^18^ He discovered that measured impedance on cells and tissue gave rise to a semicircle with a suppressed center (Figure 2). The Cole model solves for R_ECF_, R_ICF_, Cm, and exponent alpha. As depicted in Figure 3, at *R*_0_ or *R*_E_, the current flows solely through the ECF, and at *R*_INF_, the effects of Cm become insignificant, and current flows freely through the ECF and ICF (*R*_I_). Differences in cell size and shape cause variation in *R*_I_ and Cm and are accounted for by exponent alpha (i.e., the Cole model).

**FIGURE 4:**
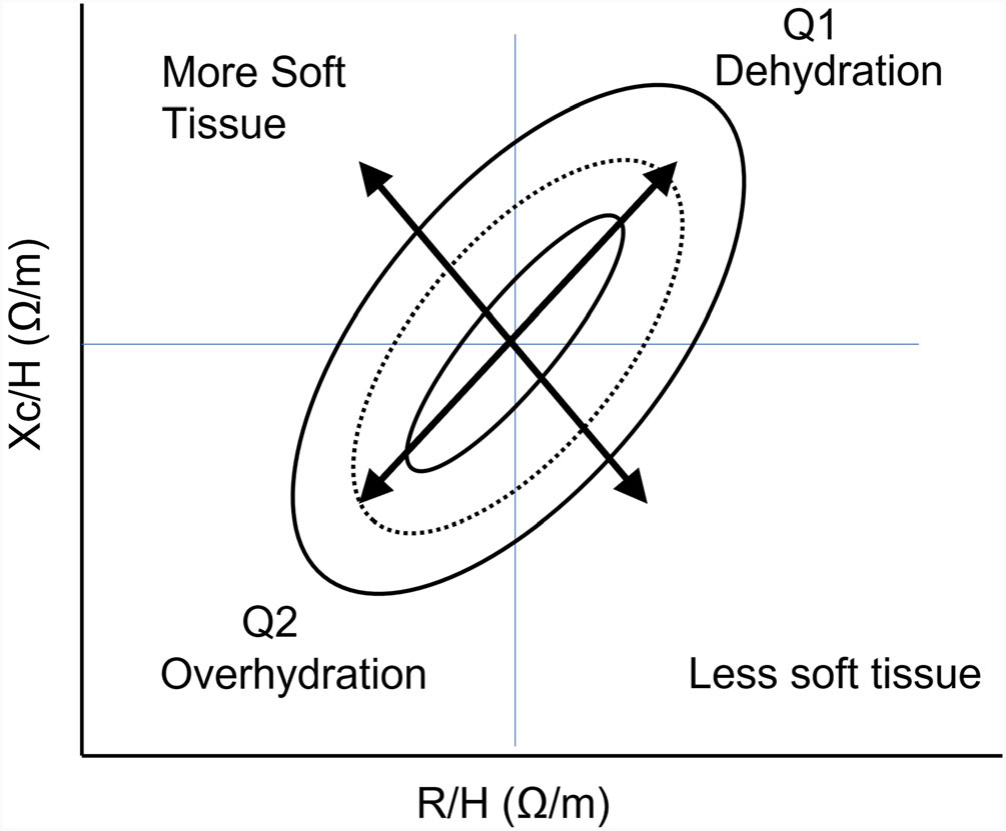
Bioimpedance vector analysis using Piccoli plot. Ellipses represent vector plots that might be expected in healthy reference population. Position and length of the vector provide information about disease status and cell membrane function. Phase angle plot in top right‐hand quadrant (Q1) is likely to be in patients that are dehydration. Plots in lower left‐hand quadrant (Q2) are likely to represent over HYD. The directional change in phase angle provides indication of change in HYD status. Adapted from Brantlov et al.^73^ [Color figure can be viewed at wileyonlinelibrary.com]

For healthy muscle tissue, the mean frequency where reactance (*X*) is maximum (called characteristic *f*; fc) is ~50 KHz. Hence, this frequency is used in simplistic SFBIA machines. The combination of the vector length and its direction is defined as the phase angle. One SFBIA 50‐KHz methodology of analysis is the Piccoli or bioimpedance vector analysis (BIVA) plot^19^ (Figure 4) which can give an indication of changes in body composition. But, there is no straightforward relationship between phase angle or BIVA and any numerical measure of volume, limiting its use in clinical practice.^20^ Moreover, fc, which is determined by ECF, ICF, and Cm, varies between individuals and changes the proportion of ICF measured at any given frequency. BIVA may have utility for population level classification, but this systematic error can only be removed at this time, by solving for Cole model terms *R*_0_ and *R*_INF_. The Cole model was a major discovery in biophysics. The ECF and ICF are significantly different materials, and its use allowed these two compartments to be accurately studied separately for the first time as: (1/*R*_ICF_ = 1/*R*_INF_ – 1/*R*_0_).

The fact that fc changes with changes in tissue hydration has important implication when SFBIA is used to estimate hydration in dialysis patients. In follow‐up to an National Institute of Health technology assessment conference, a consortium of scientists and researchers stated that “Whether additional advances can be made with SF (50 kHz) BIA measurements that will have a significant impact on routine clinical use appears less likely than that for multifrequency BIA (MFBIA) measurements.”^21^

MFBIA devices pass a signal through the body at multiple fixed frequencies in the range of 5–500 kHz. The lowest frequencies are used to quantify ECF, with higher frequencies used to predict TBW. ICF volume is then calculated from the difference of the two. BIS is a more sophisticated form of MFBIA. It uses a wider range of frequencies and the least squares curve fitting method to give a superior estimation of *R*_∞_ and *R*_0_ (and hence ECV and TBW resistance). As indicated, ECF and ICF volume are then computed.^22^

In 2007, Chamney proposed that estimating hydration status required accounting for the fluid in normally hydrated (NH) adipose tissue (~20%) and NH lean tissues (~73%) and proposed a method whereby expected ECF and ICF volume ratios in NH lean and NH adipose tissues are compared to BIS measured ECF an ICF volumes. This permits a more accurate prediction of NH lean and adipose tissue masses and reveals hydration (HYD) status.^10^ Along with the work of Moissl et al.,^22^ this is the approach the Fresenius Body Composition Monitor (BCM) uses.

Using the FMC BCM machine, 90% of normal controls have OH ± 1.1 L,^23^ but it has been found that 25% of prevalent PD patients are overhydrated by >2.5 L (OH/ECW > 15%) which is suggested as the over HYD threshold for PD populations.^24^ The InBody MFBIA devices estimate HYD status by measuring ECW/TBW. A ratio of >0.4 is suggested by the manufacturer based on measurements in 6520 normal healthy Koreans to indicate over HYD.^4^ As explained, the equations and algorithms used by these two machines to estimate intracellular water (ICW), ECW, and TBW are different, and therefore, it is unsurprising that estimated ECW and TBW are also different (in the order of 8% in HD and healthy controls).^25^ Whether this difference is of clinical relevance remains to be determined. But there is evidence that BIS guided fluid management can lead to improved HYD status,^26^ blood pressure (BP),^27^ and is independent predictor of LV systolic function in PD patients.^28^

## 3 ESTIMATING OVERHYDRATION BY BIA (OH OR ECW/TBW) IN PD PATIENTS

While BIA techniques have been reproducible and validated with gold standard dilution methods in healthy populations,^22^ there are no validation studies performed specifically in the PD population. BIS measurement values are based on algorithms derived from healthy Caucasian populations whose body composition and fluid distribution are different to PD patients.^29^ A cross‐sectional study of 40 PD patients showed wide limits of agreement between gold standard dilution methods and multifrequency BIA for TBW and extracellular volume (ECV).^30^ Moreover, cut‐off figures defining severe overhydration in PD patients ([OH/ECW] × 100 > 15–17%) were extrapolated from HD studies where HYD status above this value was associated with worse survival in multivariate Cox regression analysis specific to HD patients (HR 2.72).^31^

The algorithm to calculate overhydration in dialysis patients (and PD in particular) can be confounded by obesity^32^ and changes in body composition (fat and lean tissue mass [FTM and LTM]). The association between over HYD and markers of malnutrition has been repeatedly documented. Part of this association may be caused by bias in the algorithm when malnutrition is present; a strong predictor of discrepancy between measured and estimated TBW was plasma albumin.^33^ TBW measurements in BIA assume a fixed HYD of lean body mass^10^; however, PD patients with low serum albumin have increased tissue HYD leading to underestimates and inaccurate TBW measurements.^29^ ECW/TBW ratio is increased with muscle wasting and abnormal tissue HYD, and this is exacerbated in PD patients who often have increasing FTM while simultaneously experience loss of LTM.^29^

It is much more convenient for PD patients and staff to measure BIS with dialysate within the abdominal cavity, but Davenport reported that 2 L of dialysate in the peritoneal cavity overestimated ECW (mean difference of 0.52 L), ICW (mean difference of 0.71), and therefore trunk ECW/TBW (mean difference of 0.002).^34^ Differences in HYD measured by BCM was also confirmed in an observational study in 34 PD patients before and after draining the cavity (ΔOH was 0.18 L, p = 0.043).^35^ Others argue that whole body BIA does not measure sequestered fluid in the trunk suggesting that perhaps the “error” is in the weight entered into the BIS machine (should be the measured weight minus weight of dialysate fluid). We believe that while statistically significant, errors of this size are not clinically significant. Moreover, what is more important is consistency when performing serial measurements. Each unit should adopt a standardized method such that serial measurements are then comparable.

Another apprehension of using BIA is that achieving euvolemia may be detrimental to RRF and PD patients should be run “wet.” However, in a cross‐sectional study, we found no evidence to support the theory that reduction of over HYD when conducted with BIS monitoring was associated with increased loss of RRF.^36^ The COMPASS study, a multicenter RCT of 137 PD patients, also did not find a reduction in RRF with BIS guided fluid management.^37^

## 4 ALTERNATIVE METHODS OF MEASURING HYD STATUS

Other methods used to objectively assess fluid status in both HD and PD patients have included measurements of inferior vena cava (IVC) diameter (collapsibility index), lung ultrasound (comet lines), dual‐energy‐X‐ray absorptiometry (DXA), and biomarkers such as brain natriuretic peptide (BNP) and N‐terminal pro‐B‐type natriuretic peptide (NT‐pro‐BNP).^29^

DXA provides information on FTM, LTM, and bone tissue mass (, BTM)^38^ and is considered superior to other methods for determining body composition in dialysis patients.^39^ However, HYD status can affect estimation of lean soft tissue mass, so DXA should be combined with gold standard trace dilution methods,^39^ which limits its use in clinical practice. The risk of repeated radiation exposure also restricts its use as a monitoring tool.

Both BNP and NT‐pro‐BNP biomarkers show good correlation with echocardiographic parameters of the left ventricle^40^ but have limitations for use in volume status assessment.^40^ Both biomarkers are renally excreted and, therefore irrespective of volume status, can increase as RRF declines.^41^, ^42^ Moreover, severity of structural heart disease affects the levels of these biomarker more than the changes in fluid status.^41^, ^43^ To date, there have also been no PD studies evaluating either biomarkers against gold standard techniques.^29^ While studies^44^, ^45^, ^46^ have shown inconsistent results when assessing the relationship between volume assessment via BIS and NT‐proBNP, we suggest NT‐proBNP measurements can form part of a comprehensive clinical assessment which should include echocardiography (particularly to identify overt cardiac disease^47^) or serial BIS.

Lung ultrasound (US) can detect clinically asymptomatic pulmonary congestion, presenting hyperechoic artifacts known as lung comets lines. Recognizing pulmonary congestion has clinical value as it is an early and important clinical consequence of volume overload.^48^ However, lung comets are not specific for fluid overload and are present in other types of lung disease including interstitial pulmonary fibrosis and acute respiratory distress syndrome.^49^ The potential role of this technology was investigated in the LUST study,^50^ a recent multicenter RCT recruited 363 chronic HD patients to a 2 year study. There was good separation of number of comet lines detected between the two groups; patients in the active arm who received lung‐US guided treatment (average of 24 readings) showed a decline, whereas control patients showed an increase (p = 0.002). Post hoc analysis showed a significant reduction in repeated episodes of decompensated heart failure in the active group. However, the study did not meet statistical significance for its primary end point (all‐cause death, non‐fatal myocardial infarction, and decompensated heart failure). Failure to achieve primary endpoint may have been due to study's lack of power (enrolment only reached 77% of planned, event rate in control group was lower than predicted, and the study size was based on an expected 33% risk reduction).^50^

Only two observational studies have compared lung US to other methods of assessment in PD patients. A study from Italy compared the presence of pulmonary congestion, clinical volume overload, BIA parameters, and echocardiography in a cohort of PD patients.^51^ This showed no correlation between clinical volume overload and US findings along with significant disagreement between BIA and lung US. However, there was significant correlation of US with echocardiography parameters for volume overload.^51^ In a study of concordance between BIS measurements, lung US, and NT‐pro BNP levels, we showed a statistically significant correlation between lung US and NT‐pro BNP levels, but like the Italian cohort, there was poor correlation between US and BIS parameters.^46^ In combination, both studies suggest that biochemical markers, lung US, and echocardiography are particularly useful in identifying fluid overload in the intravascular compartment of ECV which is known to be associated with cardiovascular mortality. In contrast, BIA techniques provide information on overall HYD status as well as ICV which is directly associated with muscle mass.^52^ Biomarkers of plasma volume and cardiac function are, perhaps, complimentary techniques allowing clinicians to better understand the degree and the clinical impact of fluid overload on cardiac dysfunction. We also suggest that serial measurements will prove most useful in clinical evaluation of patients.^53^, ^54^

## 5 USE OF BIS FOR MONITORING NUTRITION (CHANGES IN FTM AND LTM)

Identifying patients with sarcopenia and malnutrition is important as both are poor prognostic indicators for patients on PD. BIS can therefore be a useful tool for risk stratifying PD patients, helping to identify patients exhibiting loss of lean muscle mass and/or obesity (high adiposity). Although DXA is not used in clinical practice for measuring LTM and FTM, it is generally considered a precise method of measurement. Studies have reported a strong correlation between lean body mass measured by BIS and DXA in PD patients.^55^

Adjusting the “raw” LTM and FTM data is important as loss of 1 kg of lean tissue, for example, is more clinically significant for a small, elderly female than a large male. The BCM machine outputs values adjusted for body size using height squared; labeled Lean Tissue Index and Fat Tissue Index. Alternatives include adjustments by weight or body mass index (BMI). We converted LTM and FTM into *z* scores reflecting the values relative to a healthy person of same age and gender, respectively.^56^ *Z* score can be derived using data available upon request to FMC which includes the mean, 90th centile LTM and FTM values for cohorts of age and gender matched subjects. Using these values, as expected, we found that low BIS measured LTM was an independent predictor of mortality while high FTM conferred a survival advantage.

Other studies using SF BIA technology, by necessity, have expressed “nutritional” status differently (ratio of extracellular mass to body cell mass or body capacitive index). These studies also confirm the relationship of “nutrition” and mortality.^57^, ^58^

Potentially BIS can be a more sensitive measure to track changes in muscle and fat mass (in PD patients, these parameters can diverge significantly) than traditional anthropometric methods.

Subjective global assessment (SGA), a commonly used clinical assessment, combines history, physical examination, and includes anthropometry.^59^ SGA is generally considered the gold standard to identify malnourished patients with protein energy wasting (PEW). It was primarily conceived to be a predictor of mortality. In this respect, it has been validated in both dialysis and non‐dialysis patients.^60^ Its ability as an accurate and sensitive clinical guide to changing nutrition status of individual patients is less clear cut. Studies have shown good correlation with SGA scores and BIS.^61^, ^62^

SGA and BIS should not be considered competing clinical assessments tools. Instead, perhaps routine BIS can complement current clinical assessments. In PD, increasing adiposity is often associated with loss of muscle mass and likely sarcopenia. BIS can differentiate the changes in body composition which can be obscured if only weight or BMI is monitored. BIS measurements are likely to be more reproducible and have lower inter‐observer variations than anthropometric assessments. It is also likely that BIS assessment will be more sensitive at detecting changes than anthropometry or SGA scores. Indeed, longitudinal change in body composition measured by BIA was found to be associated with mortality.^54^ It is also encouraging to note that a recent RCT showed an improvement of nutritional status (determined by SGA) in patients randomized to have BIS monitoring compared to control group.^63^

## 6 INTERVENTIONAL STUDIES USING BIA

Observational studies confirm that BIA in dialysis has a predictive prognostic value.^12^ In a systematic review of PD studies in 32 out of 38 studies (beware that these are a mix of different BIA technologies including SFBIA, MFBIA, and BIS), FO determined by BIA was associated with worse survival independent of age, comorbidity, and serum albumin, with mortality doubling in the top 15% of overhydrated patients.^13^ This increased risk is independent of BP and synergistic with inflammation.^13^ BIS measurements have also been associated with technique failure as well as a higher risk of peritonitis.^3^, ^5^, ^64^

However, interventional BIA studies in PD patients have been less successful than in the seminal pilot study of 131 HD patients which demonstrated that using BIS was associated with decline in arterial stiffness, systolic BP control, and reduction in all‐cause mortality.^65^ There have been five published RCT trials in PD using BIA (four used BIS, one used SF BIA) versus standard care with primary or secondary endpoint being final HYD status (as determined by BIA). The largest study recruited 308 patients using SFBIA. The largest study using BIS (240 patients) was also the most recently published. Two of four studies using BIS technology resulted in patients having a statistically significant better HYD status at the end of study.^27^, ^63^ Half the studies using BIS did not find any improvement in HYD status of intervention patients compared with controls.^37^, ^66^ The study that used SFBIA recruited patients from the United Kingdom and China was mostly negative; only anuric patients in China who had BIA monitoring had an improved HYD parameter. The improvement in ECW/TBW in anuric Chinese patients was not confirmed in the U.K. cohort or in non‐anuric patients from both countries.^67^ Cardiovascular mortality was reported in three studies, and all reported no statistical differences between control and intervention (Table 1).

**TABLE 1:** Randomized controlled studies on PD patients using BIA in assessment of volume related outcomes

Author	*N*	Study design	Duration	Type BIA device used	Type of intervention	Comparator	Main outcome and results
Filipcic et al.^25^	160 CAPD patients	Open label RCT	12 weeks	BCM, BIS	BIS assessment at baseline and every 6 weeks	Clinical assessment based on symptoms, physical examination, weight, BP	**OH volume** Intervention: 2.3 ± 2.0 to 1.7 ± 1.5 L, p < 0.05 Control: 2.2 ± 1.7 to2.5 ± 1.8, p < 0.05 *Between group comparison* *p* *≤* *0.05* **ECW/ICW** Intervention: 0.98 ± 0.16 to 0.95 ± 0.13, p < 0.05 Control: 0.97 ± 0.15 to 1.00 ± 0.14, p < 0.05 *Between group comparison p = ns*
Tian et al.^26^	151 PD patients	Open label RCT	12 months	BCM, BIS	BIS assessment at baseline and every 6 months	Clinical assessment based on symptoms, physical examination, weight, BP	**Primary End‐point was to find associations between TA‐ROH and echo parameters but no direct comparison between the 2 groups**
Tan et al.^33^	137 PD patients with urine output > 500 mL	Open label RCT	12 months	BCM, BIS	BIS assessment at baseline and every 2 months	Clinical assessment based on symptoms, physical examination, weight, BP	**OH volume** Intervention: 1.6 ± 1.8 to 1.6 ± 1.8 L, p = ns Control: 1.4 ± 1.5 to1.6 ± 1.78, p = ns *Between group comparison p = ns* **ECW/TBW** Intervention: 0.46 ± 0.03 to 0.46 ± 0.03, p = ns Control: 0.46 ± 0.03 to 0.46 ± 0.03, p = ns *Between group comparison p = ns* **End of study ROH >15%** Intervention: 22% Control: 22% *Between group comparison p = ns* **Kaplan Meier analysis:** **CV event free survival** Intervention: 3.1% Control: 8.6% *Between group comparison p = ns*
Baker et al.^59^	201 PD patients with urine output > 500 mL	Open label RCT	12 months (for OH volume and RRF) 36 months for CV events	BCM, BIS	BIS assessment at baseline and every 6 months	Clinical assessment based on symptoms, physical examination, weight, BP	**End of study OH volume** Intervention: 0.9 ± 1.4 L Control: 1.4 ± 1.9 L *Between group comparison p = ns* **End of study OH/ECW** Intervention: 5.7 ± 8.1 Control: 7.9 ± 9.8 *Between group comparison p = ns* **Kaplan Meier analysis:** **CV event free survival** Intervention: 89.8% Control: 88.7% *Between group comparison p = ns*
Tan et al.^60^	308 PD patients from the UK and China	Nested open label blinded end point RCT: patients recruited in 4 groups	12 months	BI 101 ASE, SFBIA	BIS assessment at baseline and every 3 months	Clinical assessment based on symptoms, physical examination, weight, BP	**Difference in ECW/TBW** **Group 1: Anuric, China** Intervention: 0.47 ± 0.06 to 0.48 ± 0.08 Control: 0.48 ± 0.06 to 0.51 ± 0.09 *Between group comparison not presented* **Group 2: Anuric, UK** Failure to achieve power, lack of recruitment due to falling numbers of anuric patients from the U.K. centers **Group 3: Non‐Anuric, China** Intervention: 0.48 ± 0.08 to 0.48 ± 0.07 Control: 0.47 ± 0.06 to 0.47 ± 0.06 *Between group comparison not presented, but likely ns* **Group 4: Non‐Anuric UK** Intervention: 0.44 ± 0.08 to 0.46 ± 0.06 Control: 0.46 ± 0.06 to 0.46 ± 0.07 *Between group comparison not presented, but likely ns*
Furstenberg and Davenport^55^	240 PD patients from China	Single center Open label RCT	12 months	BCM, BIS	BIS assessment at baseline and every 3 months	Clinical assessment based on symptoms, physical examination, weight, BP	**Difference in ECW** Control: 15.6 ± 3.1 L Intervention: 14.5 ± 3.2 L *Between group comparison p = 0.04* **Difference in ECW/TBW** Control: 40.4 (39.3 ± 41.3) Intervention:40.0 (39.3 ± 40.7) *Between group comparison* *p = 0.0* 3 **Kaplan Meier analysis:** **CV mortality** Log rank chi squared = 1.34 *Between group comparison p = ns*

Abbreviations: BIS, bioimpedance spectroscopy; CV, cardiovascular; ECW, extracellular water; OH, overhydration; PD, peritoneal dialysis; RCT, randomized controlled trial; ROH, relative overhydration = (OH/ECW) × 100; TA‐ROH, time averaged ROH; TBW, total body water.

A reason for the negative RCTs that use BIA to assist management of PD patients is that PD studies are generally smaller and therefore underpowered.^29^ In addition, there are practical differences between PD and HD, such as less frequent adjustment of target weight in PD compared to HD who attend more frequently, as well as greater ability to attain desired dry weight in HD.^15^, ^33^

Therefore, the challenges associated with designing interventional trials do not mean that BIS in not a useful tool to manage fluid status and nutrition. BIS provides nuanced information about changes to PD patients' HYD and body composition, and the RCT algorithms are often too simplistic. Instead, we should take heart that a prospective study showed that improvement in HYD status was also associated with lowering of serum cardiac troponin.^68^ Other observational studies of PD patients showed that sustained euvolemia was associated improvements in relevant biomarkers such as regression of LVH,^69^ BP control,^27^ and echo parameters.^28^

## 7 SUMMARY

It is important to clarify terms used in this evolving field. For a decade the fixed 50‐KHz SF method was known as BIA, but BIA is now becoming a general term for BIA technology. We believe the fixed SF method should be known as SFBIA, and the fixed multifrequency method should be known as MFBIA. BIS implies fitting the measured impedance results from a wide spectrum of frequencies to a plausible theoretical model; MFBIA can be BIS, but SFBIA and not all MFBIA machines utilize BIS methodology.^70^

To date, there is equivocal evidence to suggest that using of BIS to quantify fluid overload will lead to improved HYD status. The divergence of outcome studies may be related to the technology (we suggest BIS is superior to SFBIA and MFBIA) and to how the BIA information is interpreted and acted upon. We hope that the reader appreciates that BIS provides more information than “fluid overload.”

The uses of SGA and frailty or functional assessment (Karnofsky) scores have been adopted into clinical practice without evidence that their use will necessarily lead to improved patient outcomes. In keeping with these assessments, BIS offers another method for assessing patients' mortality risk.

The potential advantage of BIS technology is that it provides additional information about HYD and body composition. It is reproducible and is potentially more precise than other assessment methods. Other assessment methods such as lung US, cardiac biomarkers, or echocardiography can give indication of fluid overload but not in the same quantitative degree of a BIS overhydration value in liters. But it is also important to understand the limitations of the BIS estimates. Changes in HYD status are prognostically important^71^ with increasing overhydration value over time perhaps more specific and clinically significant than a single “high” reading or “stable, consistently high” readings which may be inaccurate due to the assumptions required to create the equation to estimate OH, ECW, and TBW.

While the focus on the use of BIS has been directed to optimizing fluid status, perhaps its greatest value is in risk stratification based on changes in body composition. Its correlation with mortality is not superior to SGA or functional assessment scores, but it is perhaps more sensitive to change and less reliant on single observer for anthropometry measurements; allowing us to monitor effects of treatment more effectively. It should be remembered that definition of sarcopenia also involves evidence of muscle strength and not just muscle quantity. In our clinics, body composition is expressed in *z* scores, and simultaneous hand grip strength measurements are also taken.

In further support for the BIS approach, the SOZO device from Impedimed, Ltd. has recently been given FDA Breakthrough Device Designation for a proposed indication in a renal patient population. The FDA states the designation is to help quickly address a deficiency in the current standard of care and provide an accurate measure of the fluid to remove during dialysis. They indicate that currently weight scales are used to determine the accumulation of fluid, but scales cannot account for changes in body composition, such as the muscle loss frequently seen in ESRD.

Along with new developments in BIS technology, modern electronic components have become cheaper, smaller, and faster with greatly improved noise rejection for high frequency performance. Faster low‐cost BIS devices would greatly facilitate larger scale clinical use and data collection. Cella Medical, Inc. has recently posted a preprint of an unpublished study reporting promising results comparing their new small BIS device to the FMC BCM device.^72^

BIS should be viewed as one component of a comprehensive clinical review, with the potential of being a powerful adjuvant in risk stratifying PD patients. Interpreting BIS results therefore require a complex integration of multiple factors, including OH, LTM, FTM, ΔOH, ΔLTM, and ΔFTM, as well as clinical assessments of function which could be frailty or functional capacity score with handgrip strengths. Treating patients with PD is a series of compromises. Achieving greater ultrafiltration with increasing tonicity of PD fluid risks detrimental effects on nutrition, peritoneal membrane function, and RRF. Therefore, it is unrealistic to expect a simple algorithm using BIS “pictures” will alone lead to improved clinical care. BIS might, however, provide important information that allows an experienced clinician to individualize treatment to optimize care and monitor the effect of the change.

## CONFLICT OF INTEREST

S Fan has received honoraria and travel expenses for lectures from Baxter Healthcare and Fresenius Medical Care.
